# Effect of Short-Term Increases in Feed Allowances on Growth and Sexual Development in Previously Feed-Restricted Gilts

**DOI:** 10.3390/vetsci13070677

**Published:** 2026-07-13

**Authors:** Tyler Niblett, Kimberly Williams, Marguerite Cross, Alexis Clapp, Mark Estienne

**Affiliations:** 1 Department of Animal and Food Sciences, Texas Tech University, Lubbock, TX 79409, USA; 2 Virginia Tech-Tidewater Agricultural Research and Extension Center, Suffolk, VA 23437, USAmestienn@vt.edu (M.E.)

**Keywords:** gilt, reproduction, electronic sow feeder, precision livestock farming, swine, nutritional flushing, puberty

## Abstract

Previous work demonstrates that short -term increases in feed allowances (i.e., “nutritional flushing”) can improve ovulation rate. Furthermore, precision livestock farming equipment like electronic sow feeder (ESF) systems are becoming more commonplace to enhance individual sow management in group housing. The objective of this study was to determine the effects of short-term increases in feed allowances on growth and sexual development in previously feed-restricted gilts using an ESF. Prepubertal crossbred gilts were acclimated to an ESF and allocated to one of three feeding regimens, ad libitum (AD, n = 14), restricted (RS, n = 14; 2.72 kg/d), or 2.72 kg/d, until 7 days after puberty (NF, n = 14), followed by ad libitum feeding. Estrus detection and feeding regimens commenced at 160 days of age and continued for 48 days. Reproductive tracts were harvested between 8 and 11 days after second estrus. Average daily gain was improved in NF gilts compared to RS gilts (*p* = 0.04) Ad libitum-fed gilts were less feed efficient than the other treatment groups (*p* < 0.01). Improved ovulation rate was observed for NF gilts compared to RS gilts (*p* = 0.05). Feed disappearance decreased as estrus approached for AD and NF gilts (*p* < 0.01). For the regimens herein, age at puberty onset was not impacted; however, feed conversion was improved for the level of restriction implemented. Moreover, employing ESF technology may support current estrus detection methods by monitoring feeding behavior for AD-fed gilts.

## Simple Summary

Gilt longevity and retention are essential for maintaining productivity in commercial swine herds because gilts serve as the foundational replacements for the breeding herd. Optimal management of gilts relies on adequate pubertal stimulation and nutritional management to ensure breeding occurs at second or third heat and at a body weight of approximately 135–160 kg. Breeding at first heat can result in lower litter sizes. Providing higher levels of feed during the heat cycle before breeding can improve litter size. Underweight gilts at breeding may produce less milk and exhibit poor subsequent reproductive performance. In contrast, overweight gilts require more feed to maintain over their lifetime and can be removed from the herd early for reproductive or locomotive issues. Two groups of gilts were administered low or high feed regimens. A third group received a low feed regimen until 2 weeks before their second heat when they received a high feed regimen. Feeding the high regimen of feed prior to second heat improved ovulation rate. Growth rate was similar among feeding levels, and feed intake was suppressed as gilts approached standing heat. These results highlight the importance of managing pre-breeding nutrition to support long-term reproductive success and retention in the herd.

## 1. Introduction

Annual sow culling and mortality rates in the United States are 45.6 and 12.5 percent, respectively, cumulatively representing a turnover rate approaching nearly 60% [1]. Furthermore, sows are often culled at parity 3 or 4, before producing enough pigs to recover initial investment costs [2]. Reports demonstrate that gilt removal rates prior to farrowing their first litter vary between 13 and 40 percent [3,4]. Removal reasons for gilts are most related to locomotive issues and reproductive failure, which include delayed or absence of puberty, irregular estrus cycles, and infertility. The gilt is the foundational, fundamental functional unit of a swine production system, warranting the need to optimize reproductive performance, longevity, and retention throughout her lifetime.

Typically, gilts are group-housed and fed ad libitum until they reach body weights (BW) of approximately 135–160 kg by 200 days of age, when they are transported to sow farms. For optimal reproductive performance and longevity, gilts should be bred on second estrus between 135 and 160 kg [5,6]. Lifetime productivity may be greater for gilts with moderate growth to sexual maturity and moderate BW at breeding compared to gilts that are under- or overweight at first mating, while puberty onset may be delayed at growth rates less than 0.55 kg/d [7]. Nutritional flushing may be used to recover low ovulation rates observed when limit feeding is used to control growth rates, where short term increases in feed allowances are provided prior to estrus.

With the generational pivot of implementing technology in agricultural systems, Electronic Sow Feeding (ESF) systems are being adopted by operations to improve animal management efficiency and to retain individual animal management in prevalent group housing facility designs. There are limited reports which evaluate the use of ESF stations for rearing replacement gilts. At the time of writing, this is the first report which investigates nutritional flushing via ESF technology in replacement gilts. A benefit to employing this technology is that of altering feed allocations to animals on an individual basis with defined limits, rather than requiring technicians to manually allocate feed daily. Thus, the objective of this experiment was to determine the effects of short-term increases in feed allowances on growth and sexual development in previously feed-restricted gilts using an ESF.

## 2. Materials and Methods

### 2.1. Ethics Statement

This investigation was conducted at the Virginia Tech—Tidewater Agricultural Research and Extension Center in Suffolk, VA, from August to December 2023. The protocol for this investigation was reviewed and approved by the Institutional Animal Care and Use Committee of Virginia Tech (IACUC No. 23-219).

### 2.2. Animals, Feed Regimens, and Housing Conditions

**Figure 1:**
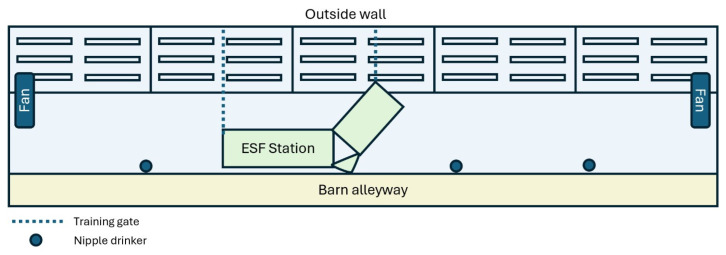
Diagram of open pen serviced by the electronic sow feeder.

Crossbred gilts (n = 42) from Berkshire × Duroc and Yorkshire × Landrace sows were employed in this study. At 150 days of age, gilts were introduced to an open pen in a curtain-sided building thatwas serviced by an ESF (Figure 1). Gilts were raised in two cohorts due to the size of the ESF pen, resulting in two replicates comprising 21 gilts each.

The pen flooring was approximately one-half solid and one-half slatted concrete. The pen contained three water nipples which provided all animals with ad libitum access to water. The ACCUTEAM G-Station (Osborne Industries, Osborne, KS, USA) was utilized in this investigation. The station was equipped with two feed hoppers and included a dedicated water line to dispense water each time feed was dispensed, providing feed as a mash. Gilts underwent acclimation and training to the ESF for 10 days during which time they received feed ad libitum. The size of the pen was 15.24 m × 3.05 m (46.48 m^2^). Stocking density was approximately 2.1 m^2^ per gilt. Gilts were introduced to the ESF at 150 ± 0.4 days of age and 92.3 ± 1.2 kg BW, and 149.7 ± 0.4 days of age and 92.6 ± 1.8 kg BW for replications 1 and 2, respectively.

### 2.3. Study Design

At 160 days of age, gilts were balanced by litter of origin and BW and randomly assigned to one of three treatments. The treatments were as follows: ad libitum (AD, n = 14), restricted (RS, n = 14), and nutritionally flushed (NF, n = 14). For replications 1 and 2, gilts were 160 ± 0.4 days of age and 102.4 ± 1.1 kg BW, and 159.7 ± 0.4 days of age and 99.8 ± 2.0 kg BW at treatment assignment. Gilts assigned to the AD group received feed on a truly ad libitum basis, and RS gilts received 2.72 kg/d. The NF group received 2.72 kg/d from the beginning of the trial (day 0) until 6 days after the initial expression of a pubertal estrus. On day 7, each NF gilt was allowed to consume feed ad libitum. This regimen was used to allow ad libitum feed access for approximately 14 days before returning to estrus. The ESF station software (TEAM version 3.3.0.19) imposes a daily limit of 5.0 kg/d of feed for animals that are considered non-productive. In this investigation, gilts thatwere allowed to consume feed ad libitum (AD group and NF group during the flushing period), were reallocated an additional 5.0 kg to allow for true ad libitum feeding. The maximum feed intake observed for gilts receiving feed ad libitum was 9.9 kg on one day for one gilt. As gilts reached second estrus, on day 0 of the subsequent cycle, feed allowances were reduced to 2.72 kg/d for all gilts, irrespective of treatment, until exsanguination.

### 2.4. Diets and Feed Delivery

Prior to the experiments and during training on the ESF, all gilts were allowed an acclimation diet on an ad libitum basis. During the experiment, animals were provided with a fortified corn and soybean meal diet. The composition of the diets is shown in Table 1.

The feed augers were calibrated weekly to ensure accurate feed allotment and BW capture. Each auger rotation dispensed approximately 0.091 kg of feed. The feed cycle began at 12:00 am and lasted for 24 h. Each day at 12:00 am, the feed cycle restarted, and gilts were allotted a new total allowable feed balance, but balances from previous days were not carried forward to the subsequent feed cycle. Each time a gilt entered the ESF, the identification number, radio frequency identification (RFID) number, entry and exit time, visit duration, amount of feed dispensed, and BW were recorded automatically as a visit observation. For this investigation, if no feed was dispensed when a gilt entered the feed station, it was classified as a non-feeding visit, and visits during which feed was dispensed were classified as feeding visits. Body weights were only captured during feeding visits. Station visits were measured on a 24 h basis as the feed cycle.

### 2.5. Estrus Detection and Reproductive Tract Harvest

Each day at 7:00 am and 7:00 pm, starting at 160 days and until 209 days, gilts were observed for physical (e.g., swollen vulva, mucous discharge) and behavioral (e.g., riding pen mates, boar seeking, altered disposition) signs of estrus in the presence of a mature boar, greater than 12 months of age. Fenceline contact with a boar occurred for approximately 20 min twice daily. Two boars were employed to reduce incidences of boar fatigue. Trained technicians mimicked tactile stimuli typically presented by the boar, including pulling at the flank, rubbing beneath the vulva, and initiating the back pressure test. Gilts which exhibited the lordosis response in the presence of the boar were recorded as in estrus. The first expression of estrus was designated as day 0 of the respective estrus cycle. Estrus duration was calculated as the difference between the first time a gilt was found in estrus and the last time she was found in estrus, based on twice daily estrus detection. If a gilt was only found in estrus at one time point, she was not included in the analysis for estrus duration. Further, if a gilt did not return to estrus before the end of the experiment, she was not included in the analysis for estrus cycle length.

Reproductive tracts were harvested 8 to 11 days after the second estrus. Gilts were weighed between 7 and 10 days after their second estrus and transported to a USDA-inspected processing plant for subsequent harvest. Tracts were removed during evisceration and kept on ice until analysis. When the first group of gilts were transported for reproductive tract harvest, a communication error occurred within the processing plant, and no tracts were collected (AD, n = 1; RS, n = 1; NF, n = 1). Therefore, only 39 reproductive tracts were collected, 27 of which had expressed a second estrus. The remaining 12 gilts were transported for reproductive tract harvest between 8 and 11 days after puberty. Whole ovaries were removed from the tract and weights were determined individually. Corpora lutea (CL) were excised from each ovary and were counted and weighed to determine an average weight. The remaining ovarian tissue was minced and blotted. Blotted ovarian tissue weight was subtracted from the ovary weight without the CL to determine follicular fluid weight. With ovaries removed, uteri were trimmed at the anterior side of the cervix to obtain uniform uterine measurements. Uteri were weighed using a benchtop lab scale. The mesometrium, mesosalpinx, and mesovarium were removed to obtain uterine horn length.

### 2.6. Growth Performance Measurements

Growth performance data was derived from the station visit data. Total feed consumption throughout the duration of the trial was summed for each gilt. Feed conversion efficiency was expressed as kg of gain per kg of feed (G:F). Average daily gain (ADG) was calculated using the formula (End weight − Start weight/Number of days on trial). Each week, beginning at day 0 of each replication, backfat (BF) thickness was measured using A-mode ultrasound (Renco Corporation, Golden Valley, MN, USA). Measurements were taken at the last rib, at the P2 position, approximately 6.5 cm from the midline.

### 2.7. Blood Collection

For each gilt, at day 0 and the day prior to exsanguination, gilts were restrained and approximately 10 mL of blood was collected via jugular venipuncture for subsequent chemistry and hematology analysis. Blood samples were distributed between tubes containing no additive for serum collection and subsequent chemistry analysis and ethylenediaminetetraacetic acid tubes for hematology analysis. Blood analyses were completed using Vetscan VS2 and HM5 analyzers (Zoetis, Parsippany, NJ, USA). For blood chemistry analysis, Comprehensive Diagnostic Profile rotors (Abaxis, Inc., Union City, CA, USA) were loaded with approximately 100 µL of serum. For hematology, each ethylenediaminetetraacetic acid tube was reinverted prior to analysis and loaded into a Vetscan HM5.

### 2.8. Statistical Analysis

Of the 42 gilts in the study, only 10 gilts from each original treatment assignment expressed a second estrus (AD, n = 10; RS, n = 10; NF, n = 10; Total, n = 30) and had reproductive tracts harvested for evaluating effects of treatment. For those gilts which only expressed a pubertal estrus (original treatment assignment: AD, n = 4; RS, n = 4; NF, n = 4; Total, n = 12), those originally assigned to the NF group were reassigned to the RS group for evaluation of station visit responses and puberty responses. These gilts did not undergo nutritional flushing from time restrictions within replications. Thus, for the single estrus gilts, the new treatment designations were AD: n = 4 and RS: n = 8. Total final treatment designations were AD, n = 14; RS, n = 18; and NF, n = 10. Analyses were conducted in subgroups including all gilts, single estrus gilts, and cycling gilts, where appropriate.

Individual gilt served as the experimental unit. Age at puberty, weight at each estrus, time in estrus, estrus cycle length, reproductive tract data, and growth performance data were analyzed by analysis of variance (ANOVA) using the MIXED procedure of SAS 9.4 (SAS Institute, Cary, NC, USA). The statistical model included the effects of feeding regimen, block within replication, and replication as possible sources of variation. The effect of feeding regimen on the proportion of gilts reaching estrus by 160, 170, 180, 190, and 200 days of age and the proportion of gilts reaching 136 kg by 200 days of age were analyzed using chi-square analysis.

Body weight and feed disappearance were processed to obtain an average BW and total feed disappearance per gilt per day. Gilts were manually weighed on the first and last day of the experiment to obtain predicted BW values to assist in removing outliers. Body weights greater than 5% from these predicted weight values were removed, as described previously [8]. Visit data (BW, feed disappearance, total station visits, feeding visits, non-feeding visits, and respective visit duration) and BF responses were analyzed by ANOVA as repeated measures using the MIXED procedure of SAS. The statistical model included feeding regimen, day of the test period, feeding regimen × day, block within replication, and replication as possible sources of variation. Day was used as the repeated effect with gilt within treatment as the subject. The autoregressive or heterogenous autoregressive covariance structures were used based on fit statistics. Visit data was combined with estrus response data to create a dataset normalized for the 20-day period prior to second estrus using PROC SUMMARY. Data was analyzed similar to the visit data with day being replaced by day of estrus in the model. Blood responses were analyzed using PROC MIXED as repeated measures with a model including effects of feeding regimen, time, block within replication, and replication as possible sources of variation [9]. The unstructured covariance structure was utilized for hematology and chemistry responses. Correlations among each chemistry and hematology response and age at puberty were evaluated using PROC CORR.

Individual means throughout were compared using the LSMEANS option with Tukey adjustments. Results are reported as least square means, and statistical differences were considered significant at *p* ≤ 0.05 with tendencies declared at 0.05 < *p* ≤ 0.10.

## 3. Results

Of the 42 gilts employed in this study, 40 (95%) of the gilts reached puberty by 200 days of age. The other two gilts reached puberty by 210 days of age. Feeding regimen did not impact the proportion of gilts reaching puberty in 10-day increments (*p* > 0.05); however, at 180 and 190 days of age, feeding regimen exerted a weak tendency (*p* = 0.12) to impact the proportion of gilts reaching puberty. Pubertal estrus responses are represented in Table 2.

On average, gilts reached puberty at 177.8 ± 1.9 days of age. While age at puberty was similar among treatments, NF gilts were observed to have reached puberty approximately 7.5 days sooner than RS gilts (181.2 vs. 173.7 days for RS and NF, respectively). Feeding regimen tended to affect pubertal estrus duration (*p* = 0.06); however, NF gilts were in puberal estrus longer when compared with RS gilts (*p* = 0.02). All gilts reached a similar BW at puberty, irrespective of treatment (*p* = 0.81), and pubertal BW was strongly (*r* = 0.73, *p* < 0.01) correlated with age at puberty.

Data was subsequently analyzed on a puberty-only or return-to-estrus basis, where gilts that did not return to estrus before the end of the study were analyzed separately from gilts that returned to estrus. When data from animals which completed one estrus cycle were analyzed, age at puberty was not impacted by feeding regimen; however, feeding regimen continued to impact pubertal estrus duration. Nutritionally flushed gilts were in pubertal estrus longer than RS gilts (*p* = 0.02), with AD gilts observed to have intermediate values not different from the other treatments.

**Figure 2:**
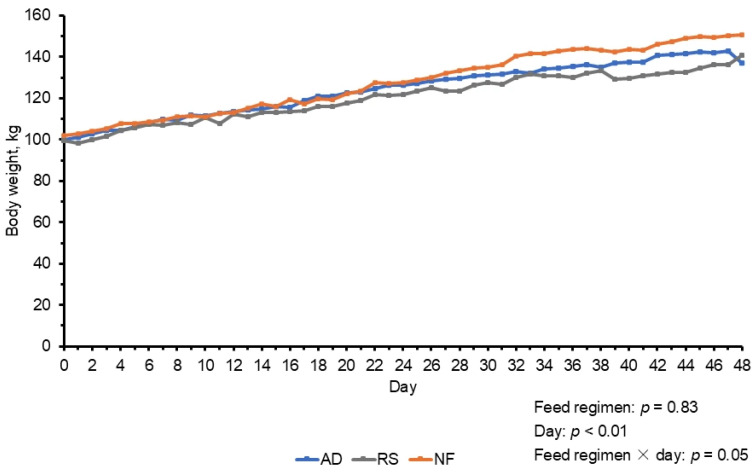
Mean body weights for previously feed restricted gilts allocated short term increases in feed allowances using an electronic sow feederfor 48 days.

Station visit response data is presented in Table 3. Throughout the trial, BW were impacted by feeding regimen × day, indicating that gilts grew at different rates (Figure 2; *p* = 0.05). Total feeding visits were impacted by feeding regimen × day, where gilts consumed feed from the ESF fewer or more times on a given day, among feeding regimens. Feeding visits were also impacted by feeding regimen, where AD gilts consumed feed from the ESF the greatest number of times (2.76 vs. 1.83 vs. 2.37, for AD, RS, and NF, respectively; *p* = 0.01). On average, gilts fed AD spent less time consuming feed per visit than RS or NF gilts (*p* = 0.03). Non-feeding visit duration tended to be different by day among feeding regimens, indicated by a feeding regimen × day tendency (*p* = 0.08).

**Figure 3:**
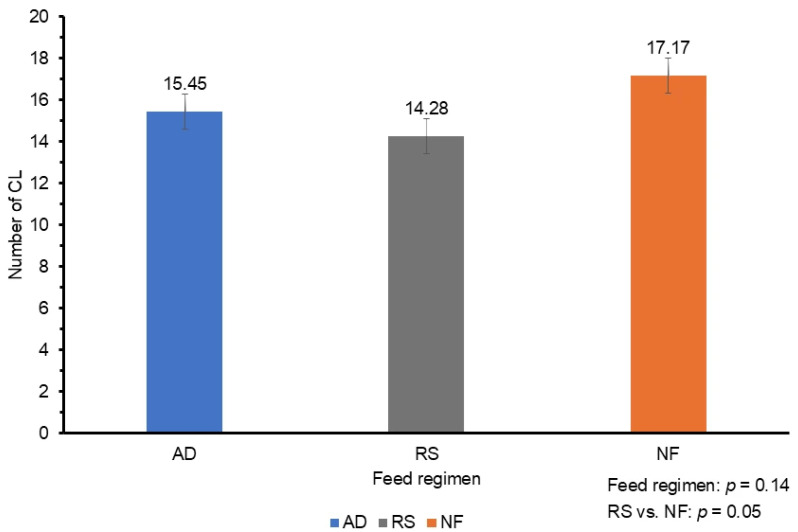
Ovulation rate for previously feed-restricted gilts allocated short-term increases in feed allowances using an electronic sow feeder.

For reproductive tracts, many observed responses were similar among treatments, shown in Table 4. Feeding regimen was observed to have a weak tendency to affect total CL (*p* = 0.14), where NF gilts were observed to have a numerically greater ovulation rate compared to other treatments, indicated in Figure 3.

With this finding, we conducted orthogonal contrasts to further investigate. Gilts subject to the NF treatment were observed to have an increased ovulation rate compared to the RS gilts (17.17 vs. 14.28; Cohen’s d = 0.96; *p* = 0.05). Ad libitum gilts were observed to have responses intermediate of and similar to the average of the other treatments (15.45 vs. 15.73, *p* = 0.79).

**Figure 4:**
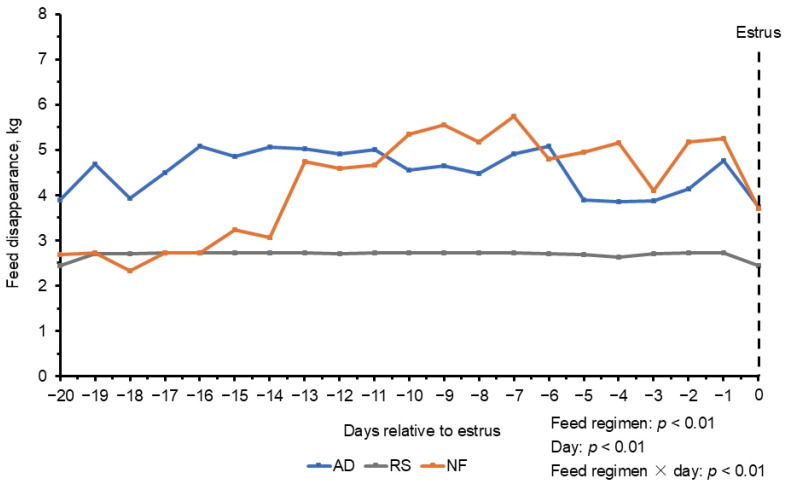
Feed disappearance of previously feed-restricted gilts allocated short-term increases in feed allowances using an electronic sow feeder, normalized for 20 days prior to estrus. Dashed line indicates the onset of estrus.

Data were subject to normalization across the 20-day period prior to second estrus. Feed disappearance was impacted by feeding regimen × day, feeding regimen, and day of estrus (all *p* < 0.01; Table 5), where feed disappearance was different among treatments daily. As estrus approached, AD and NF gilts were observed to reduce feed intake, beginning approximately 5 and 6 days prior to estrus, respectively. These patterns can be visualized in Figure 4. Throughout estrus, RS gilts visited the ESF more often than NF, and NF gilts visited the feeder more often than AD gilts (*p* < 0.01), and total visits varied by day (*p* = 0.05). Feeding visits were also greater for AD and NF gilts, compared to RS gilts (2.85 vs. 2.86 vs. 1.74; *p* < 0.01).

As BW captured by the ESF were processed to remove outliers based on predicted BW, some animals may not have full data available, especially if gilts only encountered one feeding visit. Therefore, the number of gilts for which responses were included in the analysis are denoted below each treatment mean, within response variables. The proportion of gilts reaching 136 kg by 200 days of age was similar among feeding regimens (*p* = 0.29). As expected, average daily feed intake (ADFI) was different for all treatments (4.18 vs. 2.68 vs. 3.61 kg/d for AD, RS, and NF, respectively; *p* < 0.01). Despite this finding, ADG was similar among treatments, although RS gilts were observed to express a numerical reduction in ADG compared to the other treatments (0.93 vs. 0.86 vs. 0.99 kg for AD, RS, and NF, respectively). Growth curves are represented in Figure 4. Feed conversion was improved for RS and NF gilts compared with AD gilts (*p* < 0.01). Backfat responses were affected by week (*p* < 0.01), where BF increased as the study continued. For cycling gilts, BF was affected by feeding regimen where RS gilts were observed to have less BF than AD gilts, while NF gilts had intermediate values. We also found a moderately positive correlation between ADG and average weekly BF change (*r* = 0.36, *p* = 0.02).

Correlation coefficients were obtained between puberty and for each of the blood chemistry and hematology characteristics. No blood chemistry analytes were affected by feed regimen (all *p* > 0.33). Calcium and sodium levels tended to be impacted by feeding regimen × time (both *p* = 0.08). For hematology responses, there was a tendency for monocytes to be affected by feeding regimen (*p* = 0.07). In fact, NF gilts expressed greater monocytes compared to AD gilts, with RS gilts expressing intermediate values. Red cell distribution standard deviation was impacted by feeding regimen × time (*p* = 0.04). The remaining hematology responses were not impacted by feeding regimen × time or feeding regimen (remaining *p* > 0.11). Categories for degree of correlation (i.e., moderate, weak) were derived where described previously [10]. Blood urea nitrogen was moderately correlated with age at puberty (*r* = 0.43, *p* < 0.01). Glucose was weakly correlated with age at puberty (*r* = 0.28, *p* = 0.07). Remaining blood chemistry analyte correlations were either very weak or nonexistent. For hematology characteristics, the red blood cell distribution coefficient of variation and standard deviation were weakly correlated with age at puberty (*r* = 0.24, *p* = 0.13; *r* = 0.25, *p* = 0.12, respectively). Red blood cell levels were also weakly correlated with age at puberty (*r* = 0.27, *p* = 0.09). The remaining hematology characteristics were either very weakly correlated, or no correlation existed.

## 4. Discussion

Proper gilt management is vital to the profitability and sustainability of swine production operations. Furthermore, proper gilt management ensures efficient production of service-eligible females which can enter the breeding herd to meet breeding targets and be retained in the herd for sufficient time. Regarding management, appropriate feeding is necessary to avoid overweight gilts which may be good candidates for early removal, consequent of low productivity and fertility [3,11]. Some findings reported here are consistent with our previous work which investigated the effects of AD vs. RS feeding of replacement gilts on growth and sexual development, with and without the inclusion of exogenous gonadotropins. In both investigations, we found that AD-fed gilts reduced feed consumption, typically beginning 5 days prior to the onset of estrus.

We continue to observe that implementing a limit-fed feeding regimen does not hasten or delay puberty in replacement gilts compared to that of typical ad libitum feeding currently employed until breeding in the commercial industry. Previous reports do contradict our findings; however, findings in those reports are dated, and many changes have occurred in the modernization of genetics, nutrition, etc. [12,13]. Consistency of the age at which gilts reached puberty in this report suggests that producers can aim to control growth rates of fast-growing, lean genotypes without delaying breeding eligibility. In our previous work, the difference in feeding regimens for feed restriction was calculated to be approximately 54% of the high plane of nutrition implemented. The difference in feed disappearance between the restricted and ad libitum feeding regimens here was approximately 73% of ad libitum feeding; however, the objective of this investigation was to evaluate the effects of nutritional flushing on growth and sexual development. With this in mind, the restriction applied here is not as strict as others, some which reported delays in puberty [7,14]. Additionally, reports show differences in puberty onset in the event of reduced growth rate and reduced feed consumption [15]. Others suggest that nutrient restriction alters reproductive function [16].

In terms of the nutritional flushing strategy, we report that albeit insignificant overall, ovulation rate was numerically greater in those gilts subject to a nutritional flushing feeding strategy compared to either feed restriction or ad libitum feeding. The result was an increase of 1.7 and 2.9 CL when compared to AD and RS, respectively. When comparing nutritional flushing and restricted feeding, ovulation rate was significantly greater for flushed gilts. As the common feeding strategy for replacement gilts is to provide ad libitum feed access until either a targeted weight or age are met, or first mating occurs, we investigated the differences in ovulation rate between this strategy and other regimens. We evaluated whether nutritional flushing, relative to feed restriction, corresponded with higher ovulation rates. We observed that ovulation rates between ad libitum feeding and the average of the two other regimens were similar and that different feeding regimens exerted no detriment to sexual development compared to current industry standard practices.

Comparing ovulation rates in combination with growth performance responses indicated that nutritional flushing is suggestive of a potential benefit relative to a feed restriction regimen in replacement gilts. Gilts which were flushed in the present study were observed to have similar ADG compared to ad libitum feeding, feed efficiency similar to restricted feeding, and an ovulation rate which was improved compared to the restricted gilts and numerically greater than that of ad libitum-fed gilts. Others report that when feed intake is increased from 2.1 to 3.6 kg/d, ovulation rate increased [17]. Beltranena et al. [7] found that ovulation rate could be recovered if feed intake was increased for 18 days prior to second estrus. Our flushing strategy was based on that of Zimmerman et al. [13], where 14 days of flush feeding resulted in the greatest increase in ovulation rate. We implemented this duration of nutritional flushing because the last 14 days of the estrus cycle includes the development of the progesterone-secreting CL, followed by rapid follicular development during the last 6 days of the estrus cycle. Increased feed intake has been reported to increase the metabolic clearance of progesterone [18]. As progesterone inhibits the hypothalamic–pituitary–gonadal axis, the higher plane of nutrition was expected to reduce this inhibitory action on gonadotropin secretion and release. This result may become more evident if this investigation is replicated on large-scale commercial farms.

Many reports of nutritional flushing utilize fixed higher planes of nutrition. The goal of this study was to investigate the effect of nutritional flushing with no set limit on maximum feed intake. Since the genetic potential among farms varies, this effect may be ameliorated across farms with different genetics. Modern females grow faster than their predecessors, and a challenge of introducing them to the breeding herd is controlling for a moderate growth rate. These females have continually been selected for improved ovulation rate, among other traits. Since contemporary lines have a higher inherent baseline ovulation rate, the improvement achieved by flushing may be reduced, because the physiological ceiling is closer. Alternatively, today’s females are also leaner and may rely more heavily on a nutritional stimulus to elicit the same reproductive response observed in historical genotypes. This could be due to the difference in adiposity and such hormonal signals derived from adipose tissue. Mallman et al. [17] reported that gilts which were flushed between puberty and second estrus, to be bred at third estrus, were observed to have two more viable embryos; however, continuing flushing from puberty through the third estrus yielded no benefit.

Uterine weights and horn length were found to be similar among treatments. These findings indicate that neither nutritional flushing nor restrictive feedingappear to reduce uterine development during the peripubertal period. Further, ovarian weight was not different among treatments, suggesting that feed restriction and short-term increases in feed allowances may have limited effects on ovarian mass. This indicates that reproductive advantages associated with nutritional flushing, like improved ovulation rate, are likely due to functional changes in follicular dynamics rather than changes in ovarian size.

Blood urea nitrogen is indicative of protein digestion and nitrogen catabolism and therefore is a metabolic waste product. Blood urea nitrogen was not impacted by the feeding regimens used here and were within the normal range, similar to other reports. Phenotypically, plasma urea nitrogen is negatively correlated with age at puberty as determined by Lents et al. [19], similar to our findings; however, we report a moderately positive correlation between the change in blood urea nitrogen and age at puberty, and these correlations are comparable simply because urea is freely diffused throughout cell membranes [20,21,22]. Physiological maturity is indicated by the onset of puberty, and it is also related to body composition during development. Blood urea nitrogen is coincident with lean tissue accretion and is easily a measurable index of amino acid utilization [23].

This study was conducted to collect preliminary findings from the application of ESF systems as a tool to enhance gilt development and management. Further, we evaluated the ability to provide short-term increases in feed allowances to gilts and observe the outcomes using this technology. The limited number of animals employed in this study provided initial effect-size estimates and demonstrated feasibility of the equipment used. Therefore, our responses which indicate no difference among treatments may result in an observed difference in future investigations if implemented on larger-scale production systems. As previously stated, gilts originally assigned to the NF treatment were reassigned to the RS group since they did not return to estrus prior to the end of the trial and were transported to the packing plant 8 to 11 days after first estrus. The findings reported here within a single herd warrant further investigation upon multiple, commercial-scale production systems to increase sampling. The observations herein are limited to those of peripubertal gilts, and production systems would benefit from investigations regarding the long-term effects of nutritional flushing using electronic feeding systems.

Electronic sow feeder technology allows producers to regulate feed intake of the breeding herd. Certainly, this technology could be used to control gilt growth rate to achieve targeted breeding weights. Moreover, the continuous variability of feed allotment settings allows any variety of feeding strategies to be employed. This concept is further enhanced by feeding stations which can blend or dispense multiple diets. Here, we investigated a nutritional flushing strategy that was associated with increased ovulation rate, suggesting a potential mitigation of the reduced ovulation observed witha limit-feeding strategy. We also found that based on the feeding regimens imposed here, puberty onset remains unaffected by changes in feed allocation. Considering that feed costs constitute a large portion of production costs, the facilitation of a nutritional flushing strategy helps recover ovulation rate beyond that of consistent ad libitum feeding. Further, limit feeding followed by ad libitum allowances may reduce input costs associated with feed. Monitoring feeding behavior of gilts may complement standard industry practices for general gilt management and estrus detection. In both this study and our previous work, gilts receiving high planes of nutrition consumed less feed as estrus approached.

## Figures and Tables

**Table 1:** Diet formulations and calculated analyses.

	% of Diet	
Constituents:	Acclimation	Experimental
Ground Corn	79.25	85.35
Soybean Meal	17.12	10.70
Monocalcium Phosphate	0.43	0.00
Dicalcium Phosphate	0.00	2.20
Limestone	1.18	1.00
Salt	0.30	0.50
Vitamin-TM Pre-mix	0.25	0.15
Choline Chloride	0.00	0.10
Lysine	0.30	0.00
Methionine	0.06	0.00
Threonine	0.10	0.00
Soybean Oil	0.99	0.00
Santaquin	0.02	0.00
	100.00	100.00
ME, kcal/kg	3359.42	3250.09
NE, kcal/kg	2567.45	2503.86
CP, %	15.09	12.14
Total Lysine, %	0.94	0.53
SID Lysine, %	0.84	0.44
SID, Methionine	0.28	0.19
SID, Threonine, %	0.54	0.35
SID, Cysteine, %	0.22	0.19
SID, Methionine + Cysteine, %	0.55	0.43
SID, Tryptophan, %	0.13	0.10
SID Valine, %	0.59	0.48
SID Isoleucine, %	0.56	0.43
SID Histidine, %	0.36	0.29
Cal: Phosphorous	1.35	1.34
Methionine: Lysine	33.3	43.2
Methionine + Cysteine: Lysine	59.7	86.8
Threonine: Lysine	64.4	79.9
Tryptophan: Lysine	15.7	22.5
Valine: Lysine	70.0	108.2
Isoleucine: Lysine	66.8	97.7
Histidine: Lysine	42.4	66.3
Ca, %	0.57	0.96
P, %	0.42	0.71
STTD phosphorous	0.19	0.43

ME, metabolizable energy; NE, net energy; CP, crude protein; SID, standardized ileal digestibility.

**Table 2:** Age at puberty and first estrus responses for previously feed restricted gilts allocated short term increases in feed allowances using an electronic sow feeder.

	Feed Regimen ^1^				*p*-Value ^2,3^
Item:	AD	RS	NF	SE ^4^	Feed Regimen
Number of gilts	14	18	10	---	---
Number of gilts showing puberty	14	18	10	---	0.99
Age at puberty, days	176.29	181.16	173.72	4.36	0.21
Time in estrus, hours (n)	33.48 (11)	29.03 (15)	41.64 (10)	5.25	0.06
Body weight at puberty, kg (n)	116.43 (13)	119.23 (16)	116.84 (9)	5.40	0.81

^1^ AD gilts were allotted feed ad libitum, RS gilts offered 2.72 kg/d, and NF gilts were restricted to 2.72 kg/d until 7 days after pubertal estrus, then allotted feed ad libitum. Number of responses reported in parentheses within response variable. Number of responses may be less than number of animals utilized due to only standing at one time point or having an outlying or missing BW at puberty. ^2^ *p*-value for main effect of feeding regimen. ^3^ Differences in means declared significant at *p* ≤ 0.05, tendencies at 0.05 < *p* ≤ 0.10. ^4^ SE = standard error of the mean. Maximum value for standard error of the means. AD, ad libitum treatment group; RS, restrict-fed treatment group; NF, nutritionally flushed treatment group.

**Table 3:** Body weights, feed disappearance, and station visits for previously feed-restricted gilts allocated short-term increases in feed allowances using an electronic sow feeder for 48 days.

	Feed Regimen ^1^				*p*-Values ^2,3^		
Item:	AD	RS	NF	SE ^4^	Feed Regimen	Day	Feed Regimen × Day
Number of gilts	14	18	10	---	---	---	---
Body weight, kg	126.49	122.98	128.88	3.72	0.83	<0.01	0.05
Feed disappearance, kg	3.93	2.68	3.43	0.12	0.02	<0.01	<0.01
Station visits, per 24 h							
Total	5.23	6.31	5.69	0.46	0.29	<0.01	0.31
Feeding	2.76	1.83	2.37	0.11	0.01	<0.01	<0.01
Non-feeding	2.49	4.44	3.30	0.48	0.61	<0.01	0.15
Visit duration, seconds							
Feeding	27.17	28.04	27.82	1.52	0.03	<0.01	0.23
Non-feeding	2.90	2.90	3.05	0.30	0.31	<0.01	0.08

^1^ AD gilts were allotted feed ad libitum, RS gilts offered 2.72 kg/d, and NF gilts were restricted to 2.72 kg/d until 7 days after pubertal estrus, then allotted feed ad libitum. ^2^ *p*-value for main effect of feed regimen, day, and feed regimen × day. ^3^ Differences in means declared significant at *p* ≤ 0.05, tendencies at 0.05 < *p* ≤ 0.10. ^4^ SE = standard error of the mean. Maximum value for standard error of the means. AD, ad libitum treatment group; RS, restrict-fed treatment group; NF, nutritionally flushed treatment group.

**Table 4:** Reproductive characteristics for cycling, previously feed-restricted gilts allocated short-term increases in feed allowances using an electronic sow feeder.

	Feed Regimen ^1^				*p*-Value ^2,3^
Item:	AD	RS	NF	SE ^4^	Feed Regimen
Number of gilts	9	9	9	---	---
Uterus					
Weight, g	530.64	494.88	509.69	46.08	0.69
Horn Length, cm (n)	126.09 (8)	124.14 (9)	124.42 (9)	12.64	0.98
Ovaries					
Total weight, g	8.60	8.64	10.70	0.79	0.18
Total Follicular Fluid Volume, g (n)	2.05 (9)	2.13 (9)	2.29 (8)	0.43	0.85
Total CL, n	15.45	14.28	17.17	1.35	0.14
Average CL weight, g	0.48	0.49	0.53	0.04	0.46

^1^ AD gilts were allotted feed ad libitum, RS gilts offered 2.72 kg/d, and NF gilts were restricted to 2.72 kg/d until 7 days after pubertal estrus, then allotted feed ad libitum. Number of responses reported in parentheses within response variable. Missing data due to errors during tract harvest. ^2^ *p*-value for main effect of feeding regimen. ^3^ Differences in means declared significant at *p* ≤ 0.05, tendencies at 0.05 < *p* ≤ 0.10. ^4^ SE = standard error of the mean. Maximum value for standard error of the means. AD, ad libitum treatment group; RS, restrict-fed treatment group; NF, nutritionally flushed treatment group; CL, copora lutea.

**Table 5:** Body weights, feed disappearance, and station visits for previously feed-restricted gilts allocated short-term increases in feed allowances using an electronic sow feeder, normalized for 20 days prior to estrus.

	Feed Regimen ^1^				*p*-Values ^2,3^		
Item:	AD	RS	NF	SE ^4^	Feed Regimen	DOE	Feed Regimen × DOE
Number of gilts	10	10	10	---	---	---	---
Body weight, kg	126.60	117.70	129.06	6.27	0.17	<0.01	0.08
Feed disappearance, kg	4.47	2.71	4.20	0.13	<0.01	<0.01	<0.01
Station visits, per 24 h							
Total	3.40	5.84	4.25	0.39	<0.01	0.05	0.39
Feeding	2.85	1.74	2.86	0.16	<0.01	<0.01	<0.01
Non-feeding	0.59	4.15	1.34	0.39	<0.01	0.03	<0.01
Visit duration, minutes							
Feeding	26.33	29.17	27.46	1.64	0.20	<0.01	0.57
Non-feeding	3.22	2.87	3.68	0.56	0.28	0.95	0.50

^1^ AD gilts were allotted feed ad libitum, RS gilts offered 2.72 kg/d, and NF gilts were restricted to 2.72 kg/d until 7 days after pubertal estrus, then allotted feed ad libitum. ^2^ *p*-values for main effect of feed regimen, day of estrus (DOE), and feed regimen × DOE. ^3^ Differences in means declared significant at *p* ≤ 0.05, tendencies at 0.05 < *p* ≤ 0.10. ^4^ SE = standard error of the mean. Maximum value for standard error of the means. DOE, day of estrus; AD, ad libitum treatment group; RS, restrict-fed treatment group; NF, nutritionally flushed treatment group.

## Data Availability

The data presented in this study are available on request from the corresponding author.

## Abbreviations

The following abbreviations are used in this manuscript:

AD	Ad libitum treatment group
ADFI	Average daily feed intake
ADG	Average daily gain
ANOVA	Analysis of variance
BF	Backfat
BW	Body weight
CL	Corpora lutea
DOE	Day of estrus
ESF	Electronic sow feeder
G: F	Gain to feed ratio
ME	Metabolizable energy
NE	Net energy
NF	Nutritionally flushed treatment group
RS	Restrict-fed treatment group
SID	Standardized ileal digestibility
